# *Chenopodium album* is a weed host of *Meloidogyne incognita* (Nematoda: Meloidogynidae) in Peru

**DOI:** 10.21307/jofnem-2020-099

**Published:** 2020-10-21

**Authors:** Jorge Airton Gómez-Chatata, Teodocia Gloria Casa-Ruiz, Juan José Tamo-Zegarra, Cristiano Bellé

**Affiliations:** 1 Universidad Nacional de San Agustin de Arequipa, Arequipa, Peru; 2 Universidade Federal de Santa Maria, Rio Grande do Sul, Santa Maria, Brazil; 3 Instituto Phytus, Estação experimental de Itaara, Itaara, Rio Grande do Sul, Brazil

**Keywords:** Detection, Diagnosis, Fat-hen, Identification, Root-knot nematodes

## Abstract

*Chenopodium album* plants showing symptoms caused by root-knot nematodes were detected in the La Joya, Arequipa, Peru. Based on the morphological, esterase phenotypes, and molecular analyses of the mitochondrial DNA region between the cytochome oxidase subunit II and 16S rRNA genes (mtDNA) and species-specific characterized amplified region, the causal agent of the observed symptoms was identified as *Meloidogyne incognita*. Pathogenicity was confirmed by fulfilling a modified version of Koch’s postulates. To our knowledge, this is the first report of *M. incognita* parasitizing *C. album* in Peru.

*Chenopodium album* L. (fat-hen) is cosmopolitan, annual weed species of notable economic importance. Their unique biological features, including high reproductive capacity, seed dormancy, high persistence in the soil seed bank, the ability to germinate, and grow under a wide range of environmental conditions and abiotic stress tolerance, help these species to infest diverse cropping systems (Bajwa et al., 2019). The *C. album* infest many agronomic crops and may cause >90% loss in crop yields (e.g. soybean, wheat, barley, maize, quinoa, potato, sugarbeet, sugarcane, and peanut) (Bajwa et al., 2019).

*C. album* is more problematic than other species of the genus, as the is more widespread and infests more number of crops, and it also acts as an alternate host of several crop pests and pathogens (Bellé et al., 2019). In this context, several weed species have been reported to host root-knot nematodes (*Meloidogyne* spp. Göldi, 1887). This genus of root-knot nematodes has the largest impact on major crops in the world, in addition to being the species most commonly found parasitizing weed roots (Ferraz et al., 1978; Moens and Perry, 2009; Bellé et al., 2017). In Peru, there are almost no studies reporting that weeds present in agricultural areas are natural hosts of the nematode *Meloidogyne* genus (Bazán, 2013).

In February 2020, samples of *C. album* plants collected (16°27′43.5″S; 71°49′19.6″W) within the La Joya, Arequipa Province, Peru, exhibiting many galls and egg masses due to infection by *Meloidogyne* sp. (Fig. 1). In order to identify the plant-parasitic nematode species infecting roots of these *C. album* plants, a combination of morphological, biochemical, and molecular analyses were employed.

**Figure 1:**
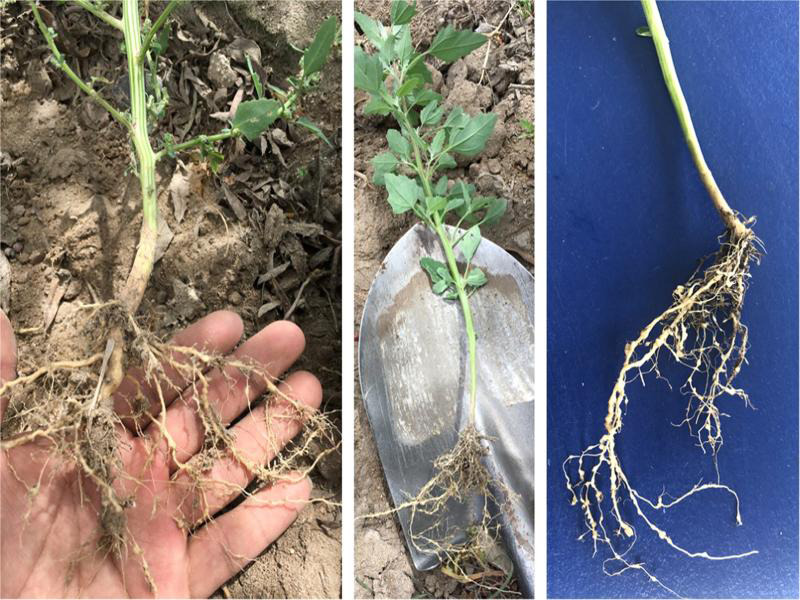
*Meloidogyne incognita* (Kofoid and White, 1919; Chitwood, 1949) root infestation symptoms on fat-hen (*Chenopodium album* L.).

The identification to species level of *Meloidogyne* population was carried out using morphological measurement of second-stage juveniles (J2) (*n* = 20), females (*n* = 20), and perineal patterns (*n* = 20 females), esterase phenotypes (*n* = 36 females), and molecular characterization of the mitochondrial DNA region between the cytochome oxidase subunit II (COII) and 16S rRNA genes (mtDNA) using the primers C2F3 and 1108 (Powers and Harris, 1993); along with PCR species-specific characterized amplified region (SCAR) sequence for confirmation, using a primer set composed of inc-K14-F and inc-K14-R (Randig et al., 2002).

The nematode population density observed in the sample was 880 J2s/g of *C. album* root. Perineal patterns of females had a high dorsal arch with wavy striae bending toward the lateral lines and the absence of distinct lateral line incisures (Fig. 2A, B). Morphological measurements of females included, body length (*L*) = 850.5 ± 20.2 (710.7-925.7) μm, stylet length = 15.1 ± 0.8 (13.5-16.9) μm, and dorsal esophageal gland orifice (DGO) = 3.8 ± 0.1 (3.2-5.2) μm. For second-stage juveniles: body length (*L*) = 402.5 ± 13.5 (384.2-419.5) μm, *a* = 22.0 ± 2.5 (20.0-24.5), *c* = 8.1 ± 0.9 (6.0-10.1), stylet length = 12.1 ± 0.2 (11.1–13.2) μm, dorsal esophageal gland orifice (DGO) = 2.2 ± 0.1 (1.7-2.4) μm, tail length = 41.60 ± 4.1 (37.2-48.9) μm and hyaline tail terminus = 12.5 ± 1.4 (11.6-14.7) μm. The overall morphology and morphometric of this population appears similar to that of *Meloidogyne incognita* (Kofoid and White, 1919; Chitwood, 1949).

**Figure 2:**
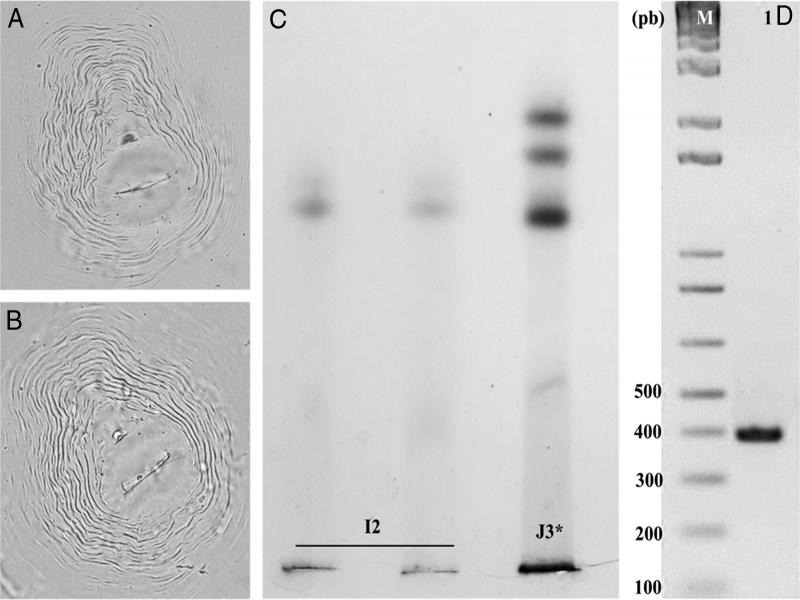
(A, B) *Meloidogyne incognita* (Kofoid and White, 1919; Chitwood, 1949) perineal patterns (scale for light microscopy photos =10 μm). (C) Esterase phenotypes of *M. incognita* detected in *Chenopodium album* L. (Est. I2 – *M. incognita* from La Joya, Arequipa Province, Peru.); J3* – *Meloidogyne javanica* reference isolate). (D) SCAR-PCR amplicons from *M. incognita* population (M: 1 kb DNA and the ladder, 1: *M. incognita* population under consideration).

The esterase phenotype (Est I2, Rm: 1.05 and 1.10) allowed for the identification of *M. incognita* (Carneiro et al., 1996) and also confirmed the purity of this population (Fig. 2C). The mtDNA sequence (e.g. 1,638 bp) was submitted to GenBank with Accession No. MT796124. Searches on BLAST showed a 99% identity with sequences of *M. incognita* from Brazil (GenBank MK861920.1), United States of America (GenBank KP001567.1 and MH152335.1), China (GenBank MH152334.1), Peru (GenBank MT066217.1), and Costa Rica (GenBank KF993635.1). To confirm the species identification, PCR using SCAR primers (Fig. 2D) amplified a specific fragment of expected size (e.g. 399 bp) typical of *M. incognita* (Randig et al., 2002).

To satisfy Koch’s postulate, *C. album* plantlets was grown in 2.5 L pots filled with a sterilized soil under greenhouse conditions. Six plantlets were inoculated with 5,000 eggs and J2s from the original population of *M. incognita*, extracted with 0.5% NaOCl according to Hussey and Barker (1973), using a blender instead of manual shaking. In addition, non-inoculated control six plants were also included in the study. Plants were maintained under greenhouse conditions at 25 ± 3°C, with watering as needed. Two months after inoculation, the root system was rinsed with tap water and weighed; then gall and egg-mass index (GI, EMI) were evaluated (Hartman and Sasser, 1985). Eggs and J2s were extracted as mentioned above, and quantified under a light microscope using Peters’ slides. The reproduction factor (RF) was calculated as RF = final population/initial population.

In this greenhouse test*, C. album* plants showed typical symptoms of *M. incognita* similar to those observed in the field. This population reproduced well in *C. album* plants, as shown by the nematode RF = 10.5, IG = 5, EMI = 5. The non-inoculated plants did not exhibit any galls. These results confirmed the pathogenicity of the *M. incognita* in *C. album* plants (Koch’s postulates).

The *C. album*, from the results obtained, is hosts of *M. incognita*, thus contributing to the maintenance and increase of populations in the field. This factor combined with competition for environmental resources, and allelopathy increase the ability of *C. album* to cause damage to crops (Raimondi et al., 2010). It is also important to note that the management of this host species is very difficult due to their extensive germination period, rapid growth, and seed viability (Heap, 2020). Thus, the difficulty in controlling *C. album* increases the complexity of *M. incognita* management in agricultural areas. However, from the knowledge of *M. incognita* polyphagous nature and its host range, an effective strategy for the management of this pathogen can be developed, reducing the damages caused to quinoa, potato, barley, wheat, maize, fruit trees, and grapevine production in Peruvian agriculture. To our knowledge, this is the first report of *M. incognita* parasitizing *C. album* in Peru.
